# Viability Reduction and *Rac1* Gene Downregulation of Heterogeneous *Ex-Vivo* Glioma Acute Slice Infected by the Oncolytic Newcastle Disease Virus Strain V4UPM

**DOI:** 10.1155/2013/248507

**Published:** 2013-03-25

**Authors:** Zulkifli Mustafa, Hilda Shazana Shamsuddin, Aini Ideris, Rohaya Ibrahim, Hasnan Jaafar, Abdul Manaf Ali, Jafri Malin Abdullah

**Affiliations:** ^1^Department of Neurosciences, School of Medical Sciences, Universiti Sains Malaysia, 16150 Kubang Kerian, Kelantan, Malaysia; ^2^Department of Pathology, School of Medical Sciences, Universiti Sains Malaysia, 16150 Kubang Kerian, Kelantan, Malaysia; ^3^Department of Microbiology, Faculty of Veterinary, Universiti Putra Malaysia, 43400 Serdang Selangor, Malaysia; ^4^Department of Biotechnology, Faculty of Agriculture and Biotechnology, Universiti Sultan Zainal Abidin, 21300 Kuala Terengganu, Malaysia

## Abstract

Oncolytic viruses have been extensively evaluated for anticancer therapy because this virus preferentially infects cancer cells without interfering with normal cells. Newcastle Disease Virus (NDV) is an avian virus and one of the intensively studied oncolytic viruses affecting many types of cancer including glioma. Nevertheless, the capability of NDV infection on heterogeneous glioma tissue in a cerebrospinal fluid atmosphere has never been reported. Recently, *Rac1 *is reported to be required for efficient NDV replication in human cancer cells and established a link between tumourigenesis and sensitivity to NDV. *Rac1 *is a member of the Rho GTPases involved in the regulation of the cell migration and cell-cycle progression. *Rac1 *knockdown leads to significant inhibition of viral replication. In this work, we demonstrated that NDV treatment led to significant reduction of tumour tissue viability of freshly isolated heterogeneous human brain tumour slice, known as an *ex vivo glioma acute slice *(EGAS). Analysis of gene expression indicated that reduced tissue viability was associated with downregulation of *Rac1*. However, the viability reduction was not persistent. We conclude that NDV treatment induced EGAS viability suppression, but subsequent downregulation of *Rac1 *gene may reduce the NDV replication and lead to regrowth of EGAS tissue.

## 1. Introduction

Glioma is a tumour of the central nervous system cancer arising from glial cells, and grade IV glioma is known as glioblastoma multiforme (GBM). GBM is the most common adult primary brain tumour with relatively low incidence compared to the other types of tumours. In Malaysia, the National Cancer Council (MAKNA) reported the incidence of brain and nervous system tumour to be 3.3 per 100. 000 people (CR) in 2006. Unfortunately, GBM showed rapid development and sustained its median survival at only 12–15 months since 25 years ago with almost 100% mortality [1–3]. Hence, insights for novel treatments have focused notably on the application of viruses, commonly known as oncolytic viruses, as antineoplastic agents.

Oncolytic viruses have been extensively evaluated, because of their potential to infect cancer cells preferentially without interfering with normal cells [1, 4–6]. NDV is one of the most intensively evaluated oncolytic viruses to affect many types of human cancer. NDV is a single-stranded negative-sense avian RNA virus in the family of Paramyxovirus that inherits selective oncolytic properties [5, 7–11]. The virus encodes for 6 viral proteins in the order of 3′-NP-P-M-F-HN-L-5′ and is divided into 3 pathotypes according to the pathogenicity in avian virus: velogenic, mesogenic, and lentogenic [11]. Two strains of NDV that are the most widely evaluated in clinical trials and have been patented for antineoplastic purposes are *HuJ* and *MTH-68*. Other strains of NDV that have been investigated are *73-T* [12], *PV-701* [13], *Ulster* [8], *Beaudette C* [9], *AF2240* [14], and *V4UPM* [15]. In particular, the V4UPM strain is a modified lentogenic or nonvirulent V4 strain that has been used as a thermostable feed pellet vaccine for poultry in Malaysia. Nevertheless, the fundamental mechanism that drives NDV infection in tumourigenic cells remains to be elucidated.

GBM cell proliferation is commonly accomplished via two signalling pathways: (1) the *Ras/Raf/ERK-MAPK/Rb* pathway and (2) the *PI3K/AKT* pathway. Currently, these pathways are attractive targets for many ongoing therapeutic trials because they are abnormally activated up to 60% in GBMs and 70% in all solid cancers [16, 17]. Example of oncolytic viruses targeting these pathways includes *reovirus* on *Ras*-activated cells, *adenovirus* on the *retinoblastoma* pathway, and *vaccinia* virus on *EGFR* [1].

In addition, another pathway that is less emphasised but has exhibited remarkable and important aberrations in GBM is the *PI3K/Rac1* pathway. This pathway controls proliferation and regulates autonomous behaviour in GBM [18, 19]. *Rac1* is a *Ras*-related C3 botulism toxin substrate 1 and is a member of the monomeric G-protein, Rho GTPases. This protein is involved in the regulation of the cell cytoskeleton and migration, gene transcription, and G1 cell-cycle progression [17, 20–22]. Gjoerup et al. reported that in an embryonic mouse fibroblast NIH3T3 cell line, activated *Rac* promoted the inactivation of *retinoblastoma* (Rb) to allow *E2F*-mediated transcription [19], thus permitting the progression of G1 into S phase of the cell cycle [23].

Recent study showed that *Rac1* was required for NDV replication in human cancer cells, and this finding established a link between tumourigenesis and sensitivity to oncolytic virus [24]. In Puhlmann et al., the dynamic siRNA approach using the skin carcinoma HaCaT cell line and *Rac1* knockdown with two different siRNAs led to significant inhibition of viral replication, thereby demonstrating that *Rac1* protein is an essential component of efficient replication of NDV in tumorigenic cells.

In this work, using the NDV strain V4UPM, we present the effects of NDV treatment on tumour viability and the *Rac1* gene expression of freshly isolated GBM slices obtained from patient in the *ex vivo* atmosphere known as EGAS. V4UPM has been previously proven to induce apoptosis in GBM cell lines [15]. In the EGAS technique, the GBM specimen was cut into round thin-slice-core and was maintained in the artificial cerebral spinal fluid (aCSF) with bubbling carbogen gas. This model aims to recapitulate the heterogeneity of GBM and its environment, which is observed in patients.

## 2. Materials and Methods

### 2.1. Virus Preparation

NDV-V4UPM was propagated as described in [25] and quantified by haemagglutination-agglutination unit (HAU) on freshly prepared young chicken red blood cells (RBC) as previously described [15].

### 2.2. Preparation of Artificial Cerebrospinal Fluid (aCSF)

Prior to tissue collection of the surgical resected tumour, aCSF was prepared according to Musshoff et al. [26]; all of the elements NaCl (124 mM/L), KCL (4 mM/L), NaH_2_PO_4_ (1.24 mM/L), MgCl_2_ (1.3 mM/L), NaH_2_CO_3_ (26 mM/L), D-Glucose (10 mM/L), and CaCl_2_ (1 mM/L) were dissolved in distilled water. The salinity at pH 7.4 ranged from 290 to 320 mM as tested by an osmometer (Osmomat 030 Genotec, Germany). The aCSF was later filtered with a 0.2 um membrane to avoid contamination of the brain slice in the organotypic culture. The sterile aCSF was later placed into the freezer to ensure that it was partially ice cold. 

### 2.3. *Ex Vivo* Acute Slice of Human Glioma Isolation and Cultivation

**Figure 1:**
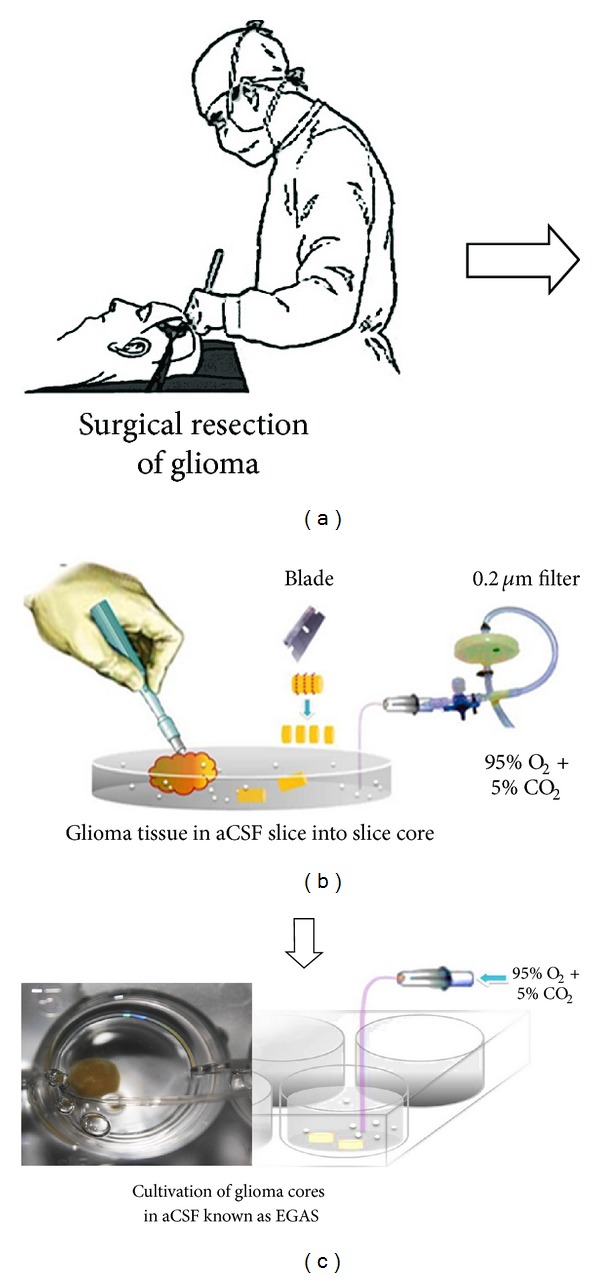
The figure summarises the protocol for preparation of EGAS cores. Surgical specimens obtained from patient (a) were immediately transferred to a petri dish containing ice-cold aCSF bubbling with filtered carbogen gas. (b) The specimen was prepared by punch biopsy and sliced with a sterile blade to form rounded glioma cores. (c) The cores were later placed into a 6-well plate for acute infection with NDV. 2 cores per group were collected from each patient (*n* = 5).

Reported by Bordey and Sontheimer, the application of the acute-slice glioma model in an aCSF had been used initially for electrophysiology analysis [27]. In our model system, a human brain tumour was obtained from patients who underwent tumour excision which was used for analysis of oncolytic viral infection. All samples were collected after a confirmed appropriate biopsy prior to surgery (ethically approved by the Research and Ethics Committee of Universiti Sains Malaysia). Next, collected tissues were immediately placed into sterile ice-cold aCSF bubbling with a mixture of 95% O_2_ and 5% CO_2_ gases known as carbogen. The carbogen was filtered through a 0.2 um filter. Following collection, the tissues were immediately transferred from the operating room to the laboratory with the carbogen being maintained. Under the sterile conditions in Biohazard Class II, the solid tissue was immersed in aCSF in a petri dish as shown in Figure 1. Several cores of the tissue were later collected by a 5 mm punch biopsy and further sectioned into a very thin core slice using a sterile blade. Sectioned slice cores were incubated for 1-hour recovery at 37°C in aCSF with bubbling carbogen, in a 6-well plate mounted on the dry bath incubator (Major Science, USA). After recovery, the cores are referred as EGAS. Later, the viability of the cores was assessed by the PrestoBlue Cell Viability Reagent as described in the following.

### 2.4. Acute Oncolytic Infection on EGAS

All collected cores were divided into three groups which are Group 1 (freshly isolated GBM and did not undergo EGAS protocol), Group 2 (mock-infected control group), and Group 3 (NDV treatment group). Following the establishment of EGAS cultivation, the method was implemented for acute *ex vivo* treatment of the glioma cores with the oncolytic NDV strain V4UPM. For the treatment group (Group 3), the infection was initiated after recovery at 37°C as shown in Figure 1(c). The infective dose (512 HAU/mL) of V4UPM for glioma [15] was mixed into the aCSF, and this treatment mixture was further incubated for another 6 hours. The same experimental procedure with virus vehicle was applied for the Group 2. At the end of the 6-hour infection, part of the infected and control cores were fixed in RNAlater (Ambion, USA) for assessment of the quantitative expression of genes by the qRT-PCR method. To analyse further the viral effects on treated EGAS viability, another core of the same group was subsequently cultivated in organotypic culture as described in the following. All results were compared to the viability of the Group 1. 

### 2.5. EGAS Tissue Viability Analysis and Organotypic Culture

The viability of EGAS cores was assessed according to the modified method reported by Diallo et al., 2010 [28], using the PrestoBlue Cell Viability Reagent (Invitrogen, USA). The reagent contains a cell-permeable blue compound that is virtually nonfluorescent but is converted to highly red fluorescent compound by the viable cells. This test is a sensitive analysis for a short incubation as compared to other viability assays. After the assay, the PrestoBlue tested tissue remains viable and can be further cultured for other assays. In our study, an EGAS core was placed in a 24-well plate with 360 uL Dulbelco's Minimum Essentials Media (DMEM) culture medium. Next, 40 uL PrestoBlue was added to the well and incubated for 30 minutes in a 37°C incubator with 5% CO_2_. After incubation, 3 samples of 100 *µ*L were collected from the well and placed into 3 wells of a 96-well plate, and absorbance was read using the spectrophotometer at 570 nm (600 nm reference wavelength for normalisation). The relative tissue viability graph was plotted according to formula viability = EGAS absorbance/fresh GBM absorbance ∗ 100. The EGAS core was transferred back into the 6-well plate for further cultivation in an organotypic culture. After 24, 48, and 72 hours, the same EGAS core viability was assessed again. For the organotypic culture, the treated and mock-treated EGAS was maintained in a 6-well plate containing 2 mL high glucose DMEM culture medium (Sigma Aldrich, USA) and incubated in the 37°C incubator with 5% CO_2_. The medium was supplemented with 10% foetal bovine serum, 1% pen-strep antibiotic, 1% nonessential amino acid (NEAA), 1 mM sodium pyruvate, and 2 mM L-glutamine, and the medium was changed daily. The core morphology was examined under an inverted microscope. The organotypic culture of brain tissues previously has been reported [29–32].

### 2.6. qRT-PCR for HN Gene of NDV-V4UPM-Infected EGAS

The qRT-PCR of all samples was performed according to the previously reported protocols [33, 34]. Briefly, RNA was extracted using the RNAqueous-Micro Kit (Ambion USA) according to the manufacturer's instructions. Total RNA yield was determined by a nanophotometer (Implen, Germany) at absorbance of 260 nm to 280 nm, and RNA integrity was determined using 1% agarose gel electrophoresis. 

For reverse transcription, single-strand cDNA synthesis was performed using Superscript III First-strand Synthesis Supermix from the qRT-PCR kit (Invitrogen, USA). The qRT-PCR analyses were performed using a probe-based detection by the ABI PRISM 7500 Sequence Detection System (Applied Biosystems, USA) and TaqMan Universal PCR Master Mix kit. The TaqMan probe contained a reporter dye at the 5′ end (FAM dye) of the probe and a quencher dye (NFQ) at the 3′ end of the probe. Intron-spinning primers and probe for all genes were designed using Primer Express Software v3.1.0 (Applied Biosystems, Germany) and synthesised by Applied Biosystems (USA) according to the GenBank entries listed in Table 1. Negative controls without reverse transcriptase were performed to ensure sufficient removal of contaminating DNA. In addition, a nontemplate control containing all reaction mixtures except the template was included to rule out contamination of the reagents. The commercially available human ACTB (GenBank Accession number NM_001101.2, Applied Biosystems, USA) with an amplicon size of 171 bp was used as endogenous reference gene. The relative expression of mRNA transcripts, standard errors and *P* values for fold-expression differences were determined by the relative expression ratio method via the Relative Expression Software Tool-Multiple Condition Solver (REST-MCS) [35].

### 2.7. Statistical Analysis

Differences of viability within experimental groups were determined by Mann-Whitney test. In addition, the relative expression ratio of target mRNA transcripts was analysed using REST-MCS software via Pair Wise Fixed Reallocation Randomisation Test. Any result with *P* < 0.05 was considered to be statistically significant. All statistical analyses were performed using SPSS 16 software.

## 3. Results

### 3.1. EGAS Core Viability Analysis by PrestoBlue Cell Viability Reagent

**Figure 2:**
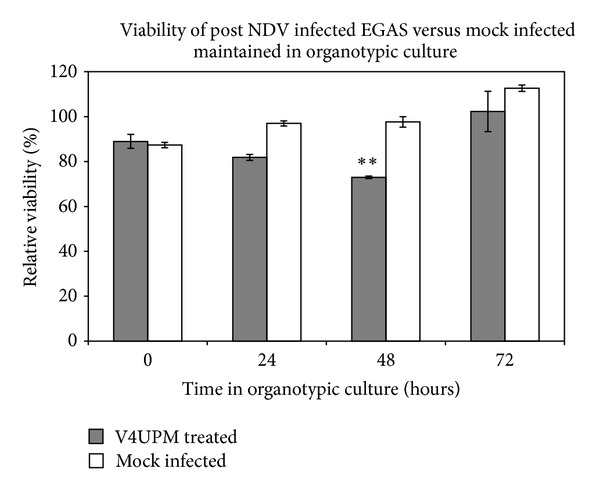
EGAS viability progression analysis and subsequent assessment in an organotypic culture following the excision from the patient and maintenance as EGAS in aCSF for 7 hours (represented as 0 hour in organotypic culture), the NDV-V4UPM treated EGAS showed a significant** reduction in the number of viable cells until 48 hours but regrowth at 72 hours, whilst mock-infected EGAS showed persistent growth with an increased number of viable cells. Error bars represent the ±SE.

To validate the use of the EGAS as an NDV target, the relative number of live cells after 7 hours of EGAS cultivation was determined by a PrestoBlue cell viability assay in DMEM medium. Compared to the viability of the Group 1, the Group 2 at 7 hours (represented by 0 hour in Figure 2) did not show a significant decrease (*P* = 0.05) in cell viability. Thus, it indicated that the post EGAS tissue remains viable throughout the EGAS cultivation. To double confirm the viability, the same tissue was subsequently cultured as organotypic culture for other 24, 48, and 72 hours. As expected, the tissue viability gradually increased in the relative number of viable cells following 72 hours in an organotypic culture. 

In the Group 3, the viability of V4UPM-infected cores after 6 hours treatment in EGAS atmosphere showed non significant reduction which were equally observed as in Group 2. Following subsequent culture, the treated tissue also showed non-significant change after 24 hours but was significantly lower at 48 hours (*P* = 0.004). Nonetheless, the viability of the infected cores was renewed after 72 hours. 

In this regard, the observation of more than 80% cell viability of mock-infected (Group 2) and treated cores (Group 3) after 6 hours demonstrated that the EGAS protocol maintains GBM core viability. However, the equal level of cell viability between both groups indicated that a 6-hour treatment did not exhibit the number of acutely dead cells. Conversely, observation of reduced viability in organotypic culture of treated cores at 24 and 48 hours demonstrated that infectivity had occurred in an EGAS treatment atmosphere. 

The regrowth of treated EGAS cores (Group 3) at 72 hours indicated that GBM replication has occurred, which coincides with our previous findings in nude mice in which treated GBMs regrew following a single treatment of V4UPM [15]. One relevant explanation of this discrepancy was described by McGinnes et al., who explained that the second generation of a nonvirulent NDV strain that proliferated in mammalian cells is not infectious and was due to lack of *furin recognition sequence* of fusion (F) protein to be proteolytically cleaved into disulphate-linked heterodimer F [25], thus forming inactive F protein. Posttranslational processing of the precursor protein F_0_ into infective F protein cleaved by a specific protease only exists in avian cells [36]. Therefore, a single dose in our treatment kills only a portion of cells, and new monocyclic V4UPM progeny produced during infection in this tumourigenic mammalian cell is not infectious. Hence, this treatment possibly has limited viral proliferation or a self-amplifying dose and led to regrowth of the tumours. Nevertheless, Puhlmann et al. has reported that *Rac1* protein is essential for efficient replication of NDV in tumourigenic cells, which kindled the interest to study the gene expression of *Rac1*. 

### 3.2. Microscopy Morphology of EGAS in Organotypic Culture

**Figure 3:**
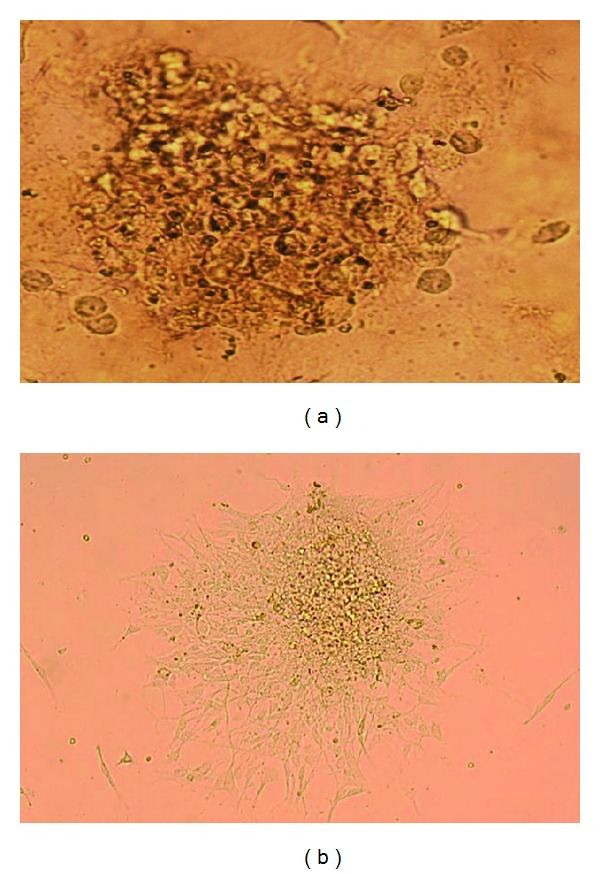
The microphotograph (10X) illustrates the infected and uninfected EGAS core after 48 hours in organotypic culture. (a) shows the treated EGAS broken into pieces and dead cells, and (b) shows the viable cells from the GBM core losing their organotypic core features, attached and spread on the culture well.

Following treatment in an EGAS atmosphere, the cores were subsequently cultivated in an organotypic culture. Parallel to viability assay, infection in Group 3 had occurred where infected cores exhibited a mix of live and dead morphology following 48 hours in culture. Nonetheless, the organotypic features were lost, and a number of live cells were also observed to migrate away from the cores (Figure 3). In contrast, mock-infected cores (Group 2) showed cell attachment, spread and continued to growth in the culture well, reflecting the increased cell progression observed in the viability analysis.

### 3.3. Gene Expression of *Rac1*, *V4UPM-HN*, and *Ras*

From the RNA extraction of the treated EGAS group (Group 3), the *V4UPM-HN* gene was expressed in all treated samples, indicating that the infection process had initiated in EGAS cores during treatment. The relative expression of mRNA transcripts of all genes that had been normalised to human *beta-actin* (ACTB) to determine pairwise fold differences in gene expression were further analysed. A previous report showed that NDV infection was a thousandfold better in *Ras*-transformed cells [37]. *Ras* is a downstream mediator of *EGF*, the pathway that is commonly activated in GBM, and leads to an increase in cell proliferation [17]. Besides *Rac1*, we also analysed the* Ras* expression in mock-infected (Group 2) and V4UPM-treated cores (Group 3). 

**Figure 4:**
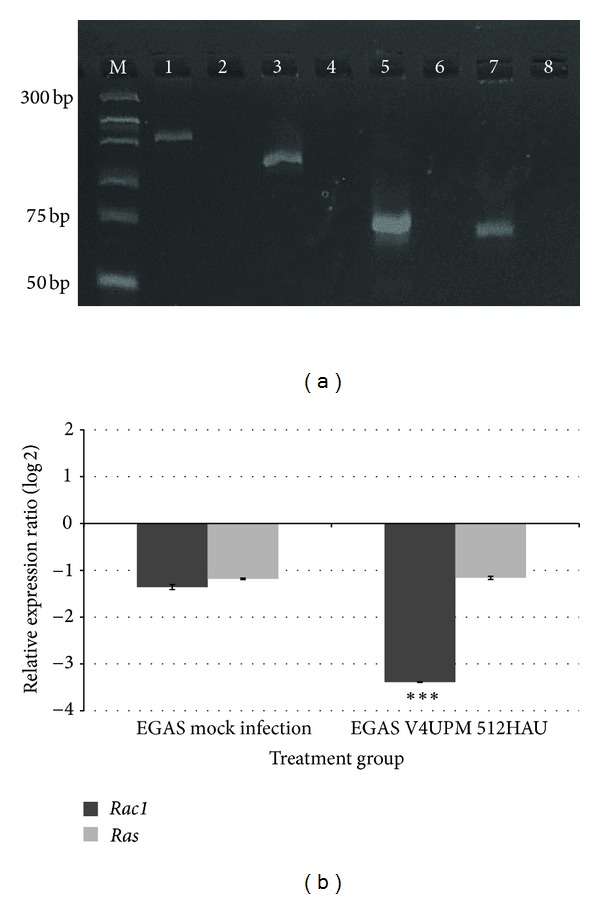
(a) SybrGreen-stained agarose gel illustrating the PCR products of human ACTB, *Rac1, V4UPM-HN, *and* Ras *genes. (M) represents the molecular weight of the marker. (1) Human ACTB 171 bp, (2) human ACTB nontemplate control (NTC), (3) *Rac1* gene 125 bp, (4) *Rac1* NTC, (5) *V4UPM-HN* gene 79 bp, (6) *V4UPM-HN* NTC, (7) *Ras* gene 70 bp, and (8) *Ras* NTC. For the *V4UPM-HN* gene, RNA was extracted from the infected EGAS, indicating the existence of viral gene in the EGAS tissue. (b) Analysis of qRT-PCR for relative expression of mRNA transcripts is determined by the relative expression ratio method via REST-MCS and the Pair Wise Fixed Reallocation Randomisation Test.

Figure 4(a) illustrates the PCR products of human ACTB, *Rac1, V4UPM-HN, *and* Ras *genes in our analysis. As shown in Figure 4(b), the *Ras* gene was not significantly downregulated at 0.440-fold and 0.447-fold in both Group 2 (*P* = 0.938) and Group 3 (*P* = 0.944), respectively, as compared to fresh GBM (Group 1). The *Rac1* gene was significantly downregulated at 0.095-fold in Group 3 (*P* = 0.001) but was not significantly changed in Group 2, with 0.390-fold changes (*P* = 0.473) having been observed.

## 4. Discussion

Despite the clinical results indicating that all oncolytic viruses in trials are safe for direct injection to the brain, overall it has fallen short of expectations [2]. NDV was the subject of relatively little preclinical investigation before being tested in clinical trials [1]. We address this issue to generate better models to compare to the conventional *in vitro* model because this incomplete achievement is associated with the shallow understanding of the barrier for oncolytic virus infection created by the wild tumour and heterogeneous tumour biology in human body. 

In this study, we are first to demonstrate the use of an EGAS for GBM-NDV acute interaction studies. The model was used to recapitulate heterogeneous features of GBM as well as to evaluate the practicality of NDV infection via CSF. Initially, we determined whether our model system supports acute NDV infection in EGAS following treatment. Previous reports showed that the NDV viral protein expression was detected after 6–8 hours [24]. From the (+) sense sequence primer design, our results indicated that expression of new *V4UPM-HN* gene was found in all treated EGAS cores. It showed that NDV infection had initiated in the aCSF environment following the 6-hour viral exposure. However, viability analysis after viral exposure did not cause any significant cell death on EGAS cores. In contrast, subsequent cultivation of the cores in an organotypic culture revealed that significant cell death only occurred after 48 hours in the treatment group, but regrowth of tumour cells was observed after 72 hours. 

NDV replicates a thousandfold more efficiently in N-*Ras* transformed cells [37]. The *Ras* gene is a downstream effector of growth factor receptors, such as *EGFR*, and is amplified in 30% of GBM [17]. In addition, a study has shown that NDV preferentially replicate in *Rac1*-expressing cell. We therefore evaluated the EGAS samples for *Ras* and *Rac1* gene expression. Our findings showed that *Ras* was slightly downregulated in both treated and untreated groups but are not statistically significant as compared to fresh GBM samples. 

In *Rac1* gene analysis, the gene was significantly downregulated in the V4UPM-treated group as compared to the control group. Our screening on NDV effects in GBM cell line by western blot indicated *Rac1* activation at 3 hours after infection (data not shown). In this regards, Puhlmann et al. had previously established a link between tumourigenesis and sensitivity to oncolytic virus via *Rac1* signalling. NDV infection preferentially replicate in *Rac1*-overexpressing cells, and inhibition of *Rac1* protein also blocked the growth of the skin carcinoma mutant cell line [24]. In cancer biology, aberrant levels of *Rac1* signalling has been implicated in many aspects such as uncontrolled proliferation [21, 22, 38], as well as growth transformation, invasion, and metastasis [18]. Specific *Rac1* gene mutations are rarely detected in GBM, but deregulated amplification is commonly found [22]. Consequently, *Rac1* appears to be an ideal target for developing approaches to block glioblastoma [3]. Earlier report had shown that suppression of *Rac1* induced apoptosis in human glioma cell but not in normal astrocytes [20]. 

Therefore, findings have pointed our study to this direction; *Rac1* activation facilitated the NDV infection, and the infection led to reduction of EGAS viability. However, subsequent *Rac1* downregulation had inhibited NDV replication and reduced its therapeutic efficiency thus leading to regrowth of EGAS viability at 72 hours. Clearly, further work has to be done to outline the specific pathway leading to GBM cell death, but NDV-*Rac1* seems to be interrelated for sustainable infection in GBM. 

### 4.1. *Ex Vivo* Glioma Acute Slice (EGAS) Model Study

The *ex vivo* model is an experiment that is performed outside of the organism with minimum alteration to the natural environment. Pertaining to brain tissue, the *ex vivo* slice model offers unique advantages over other *in vitro* or cell-culture approaches, in that they largely preserve the tissue architecture and maintain neuronal activities with intact functional local synaptic circuits for long periods of time [39]. Therefore, the use of GBM slices from patients offer great advantages, such as using heterogeneous glioma tissue, which is more relevant compared to homogeneous glioma cells in a cell-culture model. Noticeably, human tumour cell lines in culture accumulate genetic alterations during serial passaging that dramatically alter their biological behaviour [40]. In contrast, presence of normal astrocytes and necrotic cells that features the GBM possibly becomes a drawback to targeted therapy analysis.

Ideally, maintaining glioma in an acute slice preparation will provide physiologically and morphologically integrated glioma tissue for an acute infection mechanism study. The heterogeneous cells, extracellular matrix (ECM), hypoxic regions, high interstitial pressure, and higher dose requirements are amongst the major challenges in achieving lasting oncolytic viral infection [2]. As summarised in Figure 1, we used the *ex vivo* model in aCSF to maintain the heterogeneous cytoarchitecture and viability of freshly isolated human glioma tissue to study the effects of acute, oncolytic viral infection. The cerebrospinal fluid (CSF) is the colourless fluid found in the ventricle system and subarachnoid space of the brain, where it maintains the chemical stability of the brain. As observed in our viability assay, the relative number of live cells in the EGAS atmosphere after 7 hours in aCSF bubbled with carbogen gas is slightly decreased but is not significantly reduced compared to the relative number of live cells from freshly isolated GBM, thus demonstrating that the protocol has maintained the core viability (Figure 2). Therefore, the technique not only permits acute testing of the antitumour agent but also allows for temperature manipulation, oxygen deprivation (hypoxic) conditions, and higher dosages testing in the future.

## 5. Conclusions

Acute exposure of the NDV strain V4UPM to freshly isolated GBM slice in aCSF of *ex vivo* experiment had induced significant but nonlasting reduction of the slice viability. Gene expression study revealed the *Rac1* gene downregulation. This possibly reduced the NDV proliferation and its therapeutic capability, thus leading to GBM slice regrowth. Therefore, NDV-*Rac1* is interrelated in sustaining NDV tropism in glioma. 

## Figures and Tables

**Table 1:** List of sequence and GenBank accession number.

Gene	GenBank accession number	Primers sequence (5′ to 3′)	Product size
*Rac1*	NM_018890.3	Forward: TGGGATACAGCTGGACAAGA	125 bp
		Reverse: CCGATTGCCGATGTGTT	
		Probe: TATCCGCAAACAGTTGGAGAAACGT	

*Ras*	NM_176795.3	Forward: CACCAAGTCTTTTGAGGACATCCA	70 bp
		Reverse: CACGTCATCCGAGTCCTTCAC	
		Probe: ACAGGGAGCAGATCAA	

*V4UPM_HN*	X85971.1	Forward: CAGCACACCTAGCGATCTTGTAG	79 bp
		Reverse: TGGAACCGAGTGCAGATGTAATC	
		Probe: CCGACTGCGATCTCT	
